# Spiropyran-Based Drug Delivery Systems

**DOI:** 10.3389/fchem.2021.720087

**Published:** 2021-07-29

**Authors:** Andrew Fagan, Michał Bartkowski, Silvia Giordani

**Affiliations:** School of Chemical Sciences, Dublin City University (DCU), Dublin, Ireland

**Keywords:** spiropyran, merocyanine, nanocarrier, drug delivery, smart release, nanomedicine, molecular switch

## Abstract

Nanocarriers are rapidly growing in popularity in the field of drug delivery. The ability of nanocarriers to encapsulate and distribute poorly soluble drugs while minimising their undesired effects is significantly advantageous over traditional drug delivery. Nanocarriers can also be decorated with imaging moieties and targeting agents, further incrementing their functionality. Of recent interest as potential nanocarriers are spiropyrans; a family of photochromic molecular switches. Due to their multi-responsiveness to endo- and exogenous stimuli, and their intrinsic biocompatibility, they have been utilised in various drug delivery systems (DDSs) to date. In this review, we provide an overview of the developments in spiropyran-based DDSs. The benefits and drawbacks of utilising spiropyrans in drug delivery are assessed and an outline of spiropyran-based drug delivery systems is presented.

## Introduction

### Nano-Based Drug Delivery Systems

At present, many significant challenges are facing the pharmaceutical industry. Most importantly, around 40% of all drugs on the market and 60% of all new chemical entities exhibit low aqueous solubility due to the increasing size, complexity, and lipophilicity of drug molecules. Associated with this is low *in vivo* stability and toxic side effects (Fahr and Liu, 2007; Wen et al., 2015). In recent years, advances in pharmaceuticals and materials science have allowed nanomaterials to be successfully studied for their use as drug delivery systems (DDS) (Mura et al., 2013). Nanomaterials exhibit unique chemical, physical and biological properties (Krishna et al., 2018). Of particular interest is their ability to encapsulate and solubilise poorly soluble therapeutics, and release them in a controlled, target-specific manner. These drug nanocarriers exhibit prolonged retention time in the blood, improved drug efficiency and reduced side effects by limiting systemic exposure (Martinho et al., 2011). There are various organic, inorganic and metallic nanostructures used in nano-based DDSs, such as polymeric micelles, liposomes, dendrimers, and mesoporous silica nanoparticles (Deng et al., 2020).

The size of nanocarriers (1–100 nm) enables them to move more freely around the body and enhance the bioavailability of drugs due to the enhanced permeation and retention (EPR) effect (Kang et al., 2019). The EPR effect allows for the passive targeting of cells with leaky surrounding vasculatures, such as tumour cells. The functionalisation of nanocarriers’ surfaces with targeting agents allows for active targeting of specific receptor sites on target cells, thus improving the efficacy and reducing the toxic side effects of drugs (Patra et al., 2018). As a result, nanocarriers have been extensively studied for their ability to deliver anti-cancer therapeutics to specific tumour sites, such as DNA delivery vectors, and for their ability to cross the blood-brain barrier (Patra et al., 2018).

Active targeting, however, often falls short of the desired effect. Thus, to further improve the targeted and controlled release properties of nanocarriers, novel smart platforms have been developed to respond to a range of exogenous and endogenous stimuli, such as light, pH and temperature (Mura et al., 2013). This ability to control the release of drugs from nanocarriers in a time and site-specific manner offers the advantage of maximising drug efficacy while minimising side effects. Nanocarriers may be developed to respond to endogenous stimuli associated with a particular disorder, for example, the lower extracellular pH environment in tumour cells, or the significant increase in Zn^2+^ ions in apoptotic cells (Vaupel et al., 1989; Franklin and Costello, 2009). An alternative method of inducing drug release is through exogenous stimuli, such as light, magnetic fields and ultrasound. Light as a stimulus offers many advantages—it can be controlled in space and time, limiting drug release to a specific target, and the intensity and wavelength of irradiation can be precisely tuned. Light is already in use clinically for photodynamic therapy, which is used to treat cancer and vascular issues associated with angiogenesis (Moore et al., 2009). However, the wavelengths required to induce drug release, typically ultraviolet (UV) and visible (Vis) light, have poor penetration in bodily tissues, and prolonged UV-irradiation has been shown to have mutagenic effects, limiting its biomedical applications (McMillan et al., 2008; Rwei et al., 2015). These problems may be overcome using lower energy near infra-red (NIR) light, which has much greater tissue penetrating ability (Lim and Park, 2017).

Smart nanocarriers that have been developed are typically hybrid materials, such as polymers or nanoparticles, which are functionalised with a responsive material that can undergo a spatial conformational change on application of a specific stimulus (Mohapatra et al., 2019). One such class of responsive materials are spiropyrans. Spiropyrans can undergo a reversible conformational change between a closed, hydrophobic spiropyran form and an open, hydrophilic merocyanine form. This can be induced by various stimuli, such as light, pH, heat and the presence of metal ions. This unique ability to switch between two distinct, stable states on the application of a stimulus has led to their incorporation into a variety of dynamic materials (Klajn, 2014).

### The Chemistry of Spiropyrans

**FIGURE 1:**
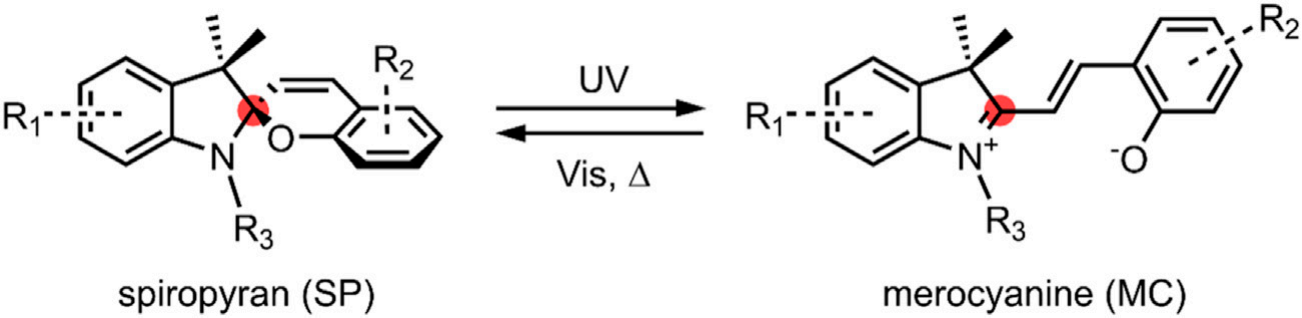
General schematic of an SP ↔ MC reversible isomerisation. The spiro-carbon is highlighted red. In the SP form, the indoline moiety on the left of the spiro-carbon is orthogonal to the benzopyran (chromene)—the MC form is planar. A strongly electron-withdrawing nitro group is typically present at the R_2_ position.

Spiropyrans are a class of organic molecules that are structurally related by the presence of a benzopyran (chromene) moiety linked to another heterocyclic moiety, commonly an indoline, *via* an sp^3^ carbon called spiro-carbon (Figure 1). The sp^3^ hybridisation of the spiro-carbon orientates the two heterocycles orthogonal to one another (Berkovic et al., 2000). This can be seen from the several available spiropyran crystal structures, and an excellent analysis of the crystal structures of three spiropyrans in the solid state, including a rare example of an open merocyanine form (Aakeröy et al., 2010).

Spiropyrans (SP) were first reported by Fischer and Hirschberg in the 1950’s, and extensively studied in the 1960’s for their photochromic behaviour (Hirshberg and Fischer, 1954a, 1954b; Heiligman-Rim et al., 1961, 1962a, 1962b). The colourless SPs become photoexcited and undergo a ring-opening upon irradiation with UV light ranging between 200–400 nm. The ring-opened intermediate that forms quickly undergoes a *cis-trans* isomerisation of the benzopyran double bond to give a stable, highly coloured open form called merocyanine (MC). The resulting MC molecule exhibits a unique absorption spectrum to the original SP molecule. This process is reversible, with the MC isomer reverting back to the more stable SP isomer under thermal conditions or by irradiation with visible light. The conversion between the SP and the MC form is not exclusively a light-responsive process. SPs exhibit acidochromism, thermochromism and solvatochromism. The MC form can also be stabilised by complexation with metal cations (Koelsch, 1951; Minkin, 2004; Kortekaas and Browne, 2019).

SP and MC are physically and chemically distinct. The SP form is neutral and non-polar. Given that the two heterocyclic moieties in the SP form are mutually perpendicular, π-electrons cannot move between them. As such, there is no delocalisation of π-electrons between the two halves of the molecule, and the molecule is not electronically conductive. As previously discussed, spiropyrans absorb in the UV region between 200–400 nm, and the absorbance spectrum is believed to be a composite of the spectra of the individual constituent moieties (Tyer and Becker, 1970). Spiropyrans are thus colourless (Eilmes, 2013; Wang and Li, 2018).

In contrast to this, upon C-O bond cleavage to the open MC form (Figure 1), a zwitterion is formed containing a phenolate anion and a positively charged indolium. The charged MCs have a much larger dipole moment (∼14–18 D) than the non-polar SP form (∼4–6 D) (Klajn, 2014). Also notable is that the spiro-carbon is sp^2^ hybridised in the ring-open form, and the indoline and benzopyran moieties are co-planar. As a result of this, there is a conjugation of the π-electrons of the two heterocyclic moieties. The conjugation present in the MC form leads to a significant redshift of the absorption spectrum compared to the SP spectrum, with the MC form absorbing in the visible region; MC is, therefore, intensely coloured (Pimienta et al., 1996; Kajimoto et al., 2010; Wang and Li, 2018). The MC form may be converted back to the SP form on irradiation with visible light, around 500–600 nm (Vlassiouk et al., 2006).

As a result of the differences between the SP and MC forms, and the ability to switch between them repeatedly by application of an external stimulus, SP molecules have received widespread attention for their use in many various dynamic systems, including SP-functionalised polymers, biopolymers, inorganic nanoparticles, carbon nanomaterials and solid surfaces—many of which have potential biological applications (Klajn, 2014; Cardano et al., 2019); notably, in cell tracking and labelling (Keyvan Rad et al., 2015; Keyvan Rad et al., 2016; Cong et al., 2021), photothermal therapy (Keyvan Rad et al., 2018) and cell sheet engineering (Karimipour et al., 2021). These applications, however, may be restricted by the well-known tendency of the MC isomer to undergo hydrolytic decomposition in aqueous solution, resulting in the formation of salicylaldehyde and a Fischer’s base derivative (Stafforst and Hilvert, 2008; Hammarson et al., 2013). This may limit the long term reversibility of SP-based molecular switches in aqueous solution. The decomposition reaction mechanism is thought to occur firstly *via* a nucleophilic attack by water on the ene-iminium MC form, followed by a retro-aldol reaction to give the decomposition products. Therefore, it has been proposed that the introduction of electron-donating groups, such as OMe, to the benzopyran moiety will greatly improve the hydrolytic stability of the MC form by increasing the electron density around the carbon-carbon double bond and reducing its susceptibility to nucleophilic attack (Abeyrathna and Liao, 2016; Berton et al., 2020). Additionally, the long-term reversibility of SP-based molecular switches is also limited by photofatigue (reduction in the efficiency of the switching process with repeat cycles). However, immobilisation of SPs to a support, such as a polymer, *via* covalent attachment at the nitrogen of the indoline moiety has been found to increase resistance to photofatigue by suppressing photodegradation pathways (Radu et al., 2009). Thus, careful tuning of the spiropyran structure and the overall system offers a successful route for overcoming these limitations.

Our group has previously shown the safety of SPs for use in biological applications; in particular, bionanosensing, where the cytotoxicity of SPs in macrophage, gastric, and epithelial cells was studied. The SPs showed negligible toxic effects on the cells up to millimolar concentrations, while significant cytotoxicity was observed only at concentrations of 10^–3^ M and above after exposure for 24 h, and at 10^–4^ M for prolonged exposure times. The time and dose dependence of cytotoxicity was also found to be statistically significant using High Content Screening and Analysis (HCSA), validated by enzyme-linked immunosorbent assays (ELISA) (Movia et al., 2010). Of recent interest is the use of SPs in nano-based drug delivery systems—several reported spiropyran-based nanocarriers are herein reviewed.

## SPIROPYRAN-BASED Nanocarriers for Drug Delivery

### Polymeric Micelles

Polymeric micelles are composed of amphiphilic block copolymers, which have distinct hydrophobic and hydrophilic domains (Zhang et al., 2014). In aqueous solutions, these amphiphilic polymers self-assemble to form micelles consisting of a hydrophobic core and a hydrophilic outer corona, with sizes ranging from 10–100 nm (Jhaveri and Torchilin, 2014). Self-assembled polymeric micelles have been studied extensively for their use in DDSs due to the enhanced solubility and circulation lifetime of the encapsulated drug, the tunability of their physicochemical properties by changing the functionalities present along the polymer backbone, and, most importantly, the EPR effect in tumours as a result of their size (Ahmad et al., 2014). The latter advantage makes them very appealing for anti-cancer drug delivery. Polymeric micelles also show promise for their use in light-controlled DDSs. Spiropyran-based micelles in which the non-polar SP form is present in the hydrophobic core surrounded by a hydrophilic shell have been recently developed. The SP form undergoes a ring-opening to form the polar MC isomer on irradiation with a specific wavelength of light. This leads to a disassembly/change in morphology of the micelle with a subsequent release of the encapsulated drug (Lee et al., 2007). This process is entirely reversible. The polymers used must be biodegradable and biocompatible. Poly (ethylene glycol) (PEG) is a commonly used hydrophilic polymer, whereas polyesters, such as poly (lactic acid), are typically used as hydrophobic polymers to make up the block copolymer backbone (Zhang et al., 2014). The light-induced isomerisation of spiropyrans makes these polymeric micelles ideal for controlled release formulations, where light offers spatiotemporal control of drug release by irradiating specific sites in the body, such as tumour cells. These systems may also be used to enhance the aqueous solubility of poorly soluble drugs, which are becoming increasingly problematic in the pharmaceutical industry (Khan et al., 2010).

**FIGURE 2:**
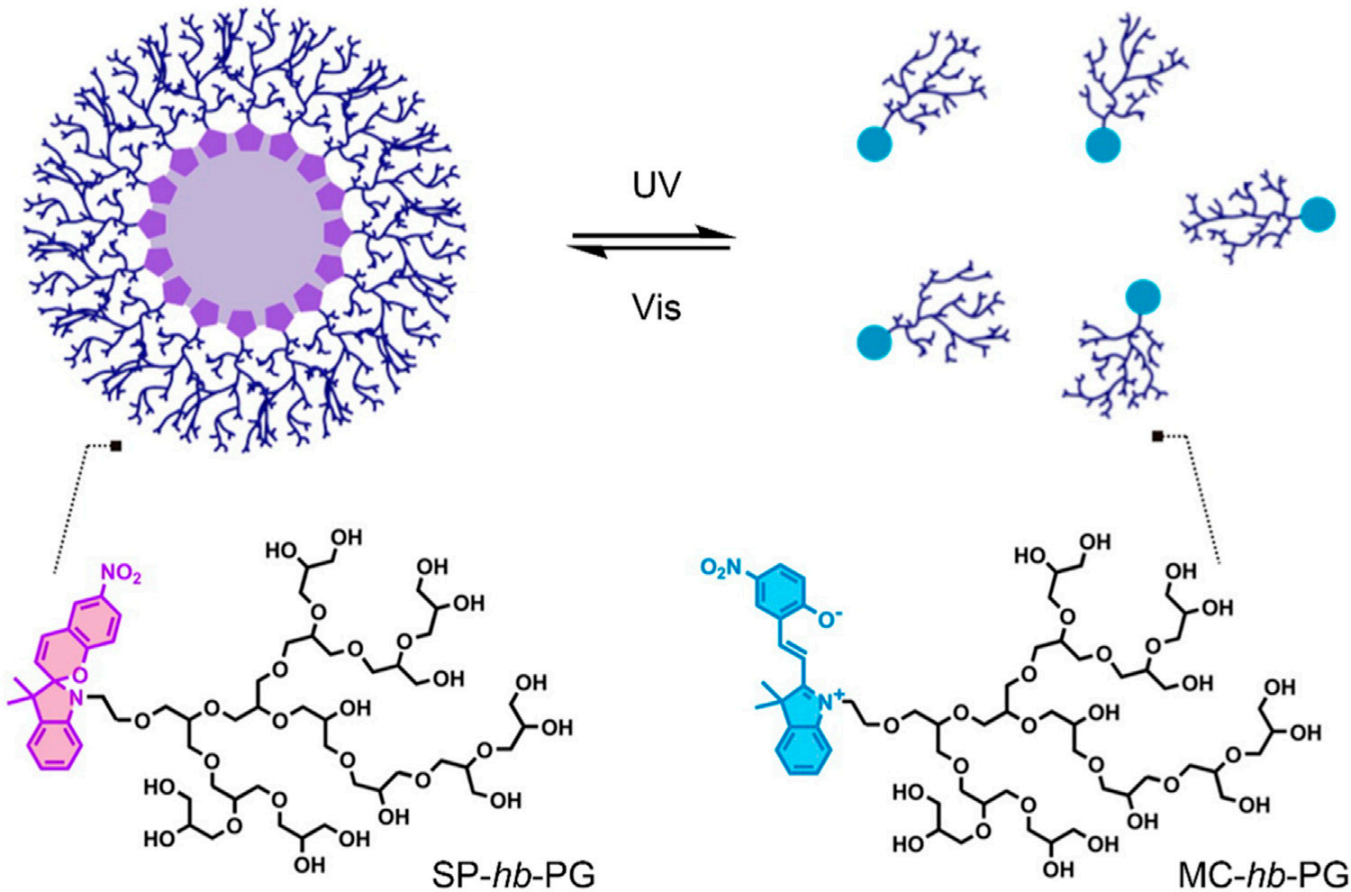
Light-induced reversible assembly of SP-*hb*-PG micelles. The SP is hydrophobic, and the MC and *hb*-PG are both hydrophilic. Figure adapted with permission from ACS (Son et al., 2014).

In 2014, Son et al. investigated the potential of amphiphilic spiropyran-initiated hyperbranched polyglycerols (SP-*hb*-PG) for light-controlled release of therapeutic agents (Figure 2) (Son et al., 2014). In solution, the amphiphilic SP-*hb*-PG chains self-assembled into micelles with an average diameter of approximately 33 nm. However, upon irradiation with UV light at 365 nm for 30 min, it was found that the diameter decreased significantly (to about 0.1 nm), suggesting the complete disassembly of the micelle into individual polymer chains. A new absorption band in the UV-Vis spectra at 550 nm, and the solutions’ colour change (from colourless to light pink), suggested that the micellar disassembly was caused by the photo-induced conversion of the amphiphilic SP-*hb*-PG to the hydrophilic MC-*hb*-PG. Upon irradiation with visible light at 620 nm for 30 min, the measured diameter returned to an average of 30 nm. This indicated the MC-*hb*-PG → SP-*hb*-PG reversion with subsequent micellar reformation, as confirmed by the disappearance of the absorption band at 550 nm in the UV-Vis spectra. The potential of this system as a nanocarrier was evaluated, using pyrene as a model hydrophobic molecule. Successful loading of pyrene into the micelle nanocarriers was achieved. The fluorescent spectrum of pyrene was observed upon irradiation with UV light at 254 nm. A decrease in fluorescence intensity with irradiation time suggested the release of pyrene from the micelles. The intensity increased again upon irradiation with visible light to about 40% of the initial intensity, suggesting partial reloading of the pyrene into the reformed micelles. *In vitro* cytotoxicity was investigated using HeLa and WI-38 cells. SP-*hb*-PG was found to be non-toxic and showed excellent biocompatibility with both cell lines (Son et al., 2014).

A similar investigation was performed using a smart DDS composed of a light-responsive amphiphilic block copolymer spiropyran-poly (2-methacryloyloxyethyl phosphorylcholine) (SP-PMPC) (Shen et al., 2015). This amphiphilic SP-PMPC system, similarly to the aforementioned SP-*hb*-PG DDS (Son et al., 2014), took advantage of the hydrophobic-hydrophilic balance in micelles. Micellar disassembly occurred upon UV-irradiation (365 nm) due to SP-PMPC → MC-PMPC isomerisation. Reassembly was observed upon visible light irradiation (620 nm), as the MC form was converted back to the SP form. Drug loading and release were also studied, using doxorubicin (DOX) as a model therapeutic. The release of DOX from the SP-PMPC micellar nanocarrier was found to increase significantly on UV-irradiation—approx. 50% of DOX was released after 24 h, whilst less than 20% was found to release in the absence of UV-irradiation after 24 h. The photo-induced on/off switching of drug release from this DDS was confirmed using the fluorescent molecule coumarin-102. Furthermore, SP-PMPC micelles were found to have no *in vitro* cytotoxic effects on huvec and HeLa cell lines, with and without UV-irradiation. Whereas the DOX loaded SP-PMPC micelles showed cytotoxicity with and without UV-irradiation. The DOX loaded micelles were also observed to have better cytotoxicity when irradiated with UV light compared to those not exposed to UV light—thus showing that the anti-cancer activity was predominantly a result of DOX release from the SP-PMPC micellar nanocarriers (Shen et al., 2015).

In 2017, Aibani et al. investigated a novel light-responsive DDS, which showed both controlled release of the loaded therapeutic agent and simultaneous real-time analysis of the quantity of therapeutic remaining in the micellar nanocarrier (Aibani et al., 2017). The micellar drug carrier was composed of an amphiphilic copolymer, which consisted of a hydrophilic PEG monomer and a hydrophobic C10 decyl chain monomer, both containing a methacrylate functional group; this amphiphilic copolymer was previously shown to be biocompatible (Yildiz et al., 2011). An SP-ibuprofen (SP-IBU) analogue was prepared and incorporated into the micelles’ hydrophobic core, along with a BODIPY dye. Upon irradiation with UV light, SP-IBU → MC-IBU isomerisation occurred, leading to the MC-IBU prodrug release from the micelle. It is believed that upon prodrug release *in situ*, the MC-IBU would undergo hydrolysis by an esterase enzyme, thus releasing the active IBU drug. The absorbance spectrum of the MC-IBU form overlaps with the emission spectrum of the BODIPY dye in the hydrophobic core, which enabled Förster Resonance Energy Transfer (FRET) to occur. FRET is a mechanism by which one donor chromophore transfers energy to another acceptor chromophore *via* dipole-dipole coupling (Helms, 2019). FRET is a distance-dependent phenomenon; the further the two chromophores are from one another, the weaker the energy transfer. In this investigation, the SP-IBU (acceptor) and BODIPY dye (donor) act as a FRET pair. When the micelles were irradiated with UV light, an SP-IBU → MC-IBU isomerisation occurred, and MC-IBU quenched the BODIPY dye’s fluorescence in the hydrophobic core due to its overlapping emission signature to that of the BODIPY. The quenching of the BODIPY dye’s emission decreased when MC-IBU was released from the core, or converted back to SP-IBU in heat and visible light conditions. This FRET communication allowed for temporal analysis of the quantity of prodrug remaining in the micelles’ core after irradiation with UV light. The system was also investigated in HeLa cells for its *in vitro* triggered encapsulated cargo release. It was shown that after 12 min, a 24 and 5% release was achieved, with and without UV-irradiation, respectively. Overall, this system shows promise for its ability to incorporate various hydrophobic APIs, thus offering a route for solubilising them. Also, drug release can be monitored by taking advantage of FRET communication between two different chromophores in the micelles’ hydrophobic core (Aibani et al., 2017).

**FIGURE 3:**
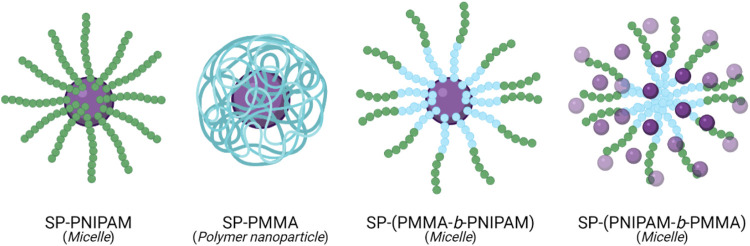
Self-assembly of SP (purple) chain end-group polymers, PNIPAM (green) and PMMA (blue), and their respective copolymers, into polymeric particles and micelles (Razavi et al., 2020).

An amphiphilic block copolymer composed of a temperature-responsive poly (*N*-isopropylacrylamide) (PNIPAM) block, and a hydrophobic poly (methyl methacrylate) (PMMA) block, with light-responsive SP chain end-groups, was investigated by Razavi et al. for its use as a dual stimuli-responsive DOX DDS (Razavi et al., 2020). Self-assembly of the block copolymer was investigated, varying the arrangement of the PNIPAM and PMMA. The particle size was found to be affected by the proximity of the SP molecule to the PNIPAM block. The composition of the micelles also varied depending on the block neighbouring the SP molecule. Micelles formed from the SP-(PMMA-*b*-PNIPAM) copolymer had a hydrophobic core containing the PMMA block and the SP molecules, and a hydrophilic corona composed of the PNIPAM block. In contrast, in micelles formed from the SP-(PNIPAM-*b*-PMMA) copolymer, the SP molecules were distributed throughout the hydrophilic corona (Figure 3). Drug release in this system resulted from the light and temperature-induced shrinkage of the micellar nanocarriers. In all self-assemblies, the SP chain end-groups underwent SP → MC isomerisation upon UV-irradiation (365 nm), resulting in a migration of the polar MC molecules to the micelles’ surface—subsequently resulting in a large decrease in particle size, inducing DOX release. This process was entirely reversible on irradiation with visible light. The SP ↔ MC isomerisation was significantly affected by the neighbouring block’s polarity, with the polar PNIPAM block stabilising the polar MC form. PNIPAM has a low critical solution temperature (LCST) range of 31–33°C. Heating the PNIPAM-based self-assemblies above the LCST (to 45°C) resulted in a significant decrease in particle size. The LCST showed light-dependence; upon UV-irradiation, the LCST increased to 37°C (close to body temperature) due to the polar MC form’s presence. This increase in the PNIPAM’s LCST is significant from a controlled-release perspective as the micelles’ temperature responsivity can be light-controlled. Moreover, the multi-responsive nature of DOX-loaded SP-PNIPAM, SP-(PMMA-*b*-PNIPAM) and SP-(PNIPAM-*b*-PMMA) micelles was investigated *in vitro*, where the release profiles were examined at pH 5.3 (25°C) and 7.4 (25°C and 40°C), and under UV irradiation at 365 nm (pH 7.4). Minimal drug release (<30%) was observed at pH 7.4 for all micellar assemblies after 48 h, whereas a significant increase in drug release was recorded in acidic media, at temperatures above the PNIPAM’s LCST (40°C), and also when irradiated with UV light. These results indicate the potential use of the micellar nanocarriers as smart DDSs, with highly efficient pH, temperature and UV light-controlled drug release. However, further investigations must be carried out to determine the ideal backbone composition for controlled release (Razavi et al., 2020).

### Polymeric Nanoparticles

In a similar fashion to micelles, amphiphilic block copolymers may self-assemble in aqueous solutions to form solid nanoparticles. The structure formed on self-assembly is dependent on the fraction of the copolymer that is hydrophilic. Polymeric nanoparticles can be classified based on their structure into nanospheres and nanocapsules. Nanospheres consist of a continuous polymeric matrix, in which a drug can be encapsulated by surface adsorption or dispersion throughout the inner matrix. Nanocapsules have a vesicular structure, composed of a hydrophobic oily core, into which hydrophobic drugs are typically dissolved, surrounded by a polymeric outer shell. As with micelles, polymeric nanoparticles have received attention as drug delivery vectors due to their favourable properties. Their small size allows for improved intracellular uptake, particularly in tumour cells due to the EPR effect. Additionally, the ability of polymeric nanoparticles to solubilise hydrophobic drugs enhances the stability, bioavailability and circulation time of the therapeutics (Begines et al., 2020; Zielińska et al., 2020). Notwithstanding the benefits of polymeric nanoparticles, one challenge associated with this nanomaterial is controlled drug release. To overcome this challenge, polymeric nanoparticles have been functionalised with SPs to developed a DDS that can deliver therapeutic agents in a controlled and site-specific manner.

In 2019, Wang et al. reported a novel pH and light-responsive polymeric nanoparticle exhibiting both dual-colour fluorescence and excellent controlled release properties (Wang et al., 2019). The nanoparticle was composed of a methyl ether poly (ethylene glycol)-poly (β-amino esters) (MPEG-PAE) copolymer, with fluorescent naphthalimide (NAPH) and photochromic SP moieties introduced along the polymer backbone *via* quaternisation. In an aqueous solution, the functionalised copolymer self-assembled to form nanoparticles with an average diameter of 40–80 nm, with the PAE, SP and NAPH composing the hydrophobic core and the MPEG forming the hydrophilic shell. On irradiation with UV light at 365 nm, there was an observable swelling and aggregation of the nanoparticles, attributed to the photo-induced conversion of the hydrophobic SP form to the hydrophilic MC form causing a disruption of the hydrophobic-hydrophilic balance in the nanoparticles. Partial reformation of nanoparticles with diameters of 10–30 nm was observed on irradiation with visible light at 520 nm, indicating reversion of the MC form back to the SP form. The SP ↔ MC isomerisation was confirmed by the appearance of an absorbance band at 560 nm, after irradiation with UV light, coupled with a yellow → purple change in colour, which is indicative of the presence of the MC form. The intensity of the absorbance band decreased again on visible light irradiation, accompanied by the reappearance of the yellow colour. The photoisomerisation also resulted in a change in the fluorescence of the nanoparticles from green to orange/red, with NAPH (donor), excited at 440 nm, and the MC isomer (acceptor) acting as a FRET pair. Acidic conditions were also found to cause changes in the nanoparticle morphology. At pH 5.5, swelling of the nanoparticles was observed, attributed to the protonation of amino groups in the PAE block, which increased its hydrophilicity. As a result of these dual stimuli responsive properties, the controlled release behaviour of the polymeric nanoparticles was investigated. The fluorescent dye coumarin-102 was used as a model hydrophobic molecule. Successful loading of coumarin-102 into the nanoparticles was confirmed by observing the fluorescence at 490 nm. A gradual decrease in the fluorescent intensity of coumarin-102 was observed on irradiation with UV light at 365 nm and pH 7, indicating the release of coumarin-102 from the nanoparticles. 83% of the loaded coumarin-102 was found to release after 35 min irradiation with UV light, while minimal release was observed in the absence of UV light. Gradual decrease in fluorescent intensity was also observed at pH 5.5, with and without UV irradiation. In the absence of UV irradiation, a release of approx. 90% was observed after 6 h, while the same release was observed after 25 min in the presence of UV light. This indicated that a combination of UV irradiation and acidic conditions could be used to achieve a rapid release of cargo from the polymeric nanoparticles, while acidic conditions alone allow for sustained release of the cargo over a longer period of time. In summary, the polymeric nanocarrier developed by Wang et al. exhibited reversible dual-colour fluorescence and dual-stimuli responsive release of a model cargo (Wang et al., 2019). However, there remains significant issues that must be addressed before the potential of this system can be fully appreciated. The loading efficiencies and biocompatibility of the nanoparticles must be examined. Additionally, the use of UV irradiation and, indeed, the irradiation times required (35 min at pH 7; 25 min at pH 5.5) challenge the clinical viability of this system given the shortcomings of using UV irradiation in a clinical setting.

### Polymersomes

As described above, the self-assembly of amphiphilic copolymers can result in the formation of micelles and nanoparticles. Polymersomes are another type of polymeric vesicles, with structures similar to liposomes; they are composed of hydrophobic bilayer membranes enclosing hollow hydrophilic cavities. This vesicular bilayer structure makes polymersomes highly attractive for their potential in encapsulation and controlled release of therapeutic agents, where hydrophobic drugs may be loaded into the hydrophobic membrane, and hydrophilic drugs may be encapsulated in the hydrophilic core (Alibolandi et al., 2015). Additionally, the ability to carefully select the constituent polymers and introduce functionalities at the supramolecular level enables a high degree of control over polymersomes’ properties; including their size, biocompatibility, and stimuli-responsiveness. Therefore, polymersomes have received widespread attention not only for their use as nanocarriers but also for their ability to mimic cellular organelles due to their bilayer membrane structure (Sun et al., 2009). The applicability of polymersomes in drug delivery is vitally dependent on membrane permeability, as the hydrophobic nature of the bilayer membrane may prevent the loading of hydrophilic drugs into the central cavity. This has led to the construction of polymersomes from stimuli-responsive polymers, enabling the modulation of properties, such as membrane permeability, so that a high degree of control over drug release can be achieved (Che and van Hest, 2016).

**FIGURE 4:**
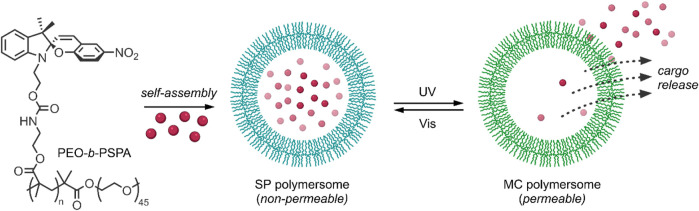
The amphiphilic diblock copolymer PEO-*b*-PSPA self-assembles into SP polymersomes, which encapsulate drug cargo in hydrophilic cavities. UV-irradiation induces switching of SP to MC polymersomes and subsequent cargo release (Wang et al., 2015).

Most methods of enhancing the membrane permeability of polymersomes, such as the introduction of stimuli-responsive bilayers, are irreversible, and the change in the hydrophobic-hydrophilic balance may lead to the undesirable disintegration of the polymersomes. To overcome this issue, Wang et al. investigated photochromic poly (ethylene oxide)-*b*-PSPA (PEO-*b*-PSPA) based polymersomes (Figure 4), composed of hydrophilic cavities encapsulated by hydrophobic PSPA (SP-based monomer with a carbamate linker) bilayers with PEO inner and outer coronas (Wang et al., 2015). The polymersomes were developed on the principle that membrane permeability could be reversibly switched between non-permeable and selectively permeable by photoinduced isomerisation between SP and MC moieties present in the membrane bilayer, respectively. On irradiation with UV light at 365 nm, the neutral SP moieties were converted into zwitterionic MC moieties, as indicated by the appearance of an absorbance band at 574 nm associated with the MC isomer. This transition was accompanied by a corresponding change in the hydrophilicity and permeability of the membrane bilayer, as indicated by a decrease in the fluorescent intensity of coumarin-102 loaded into the hydrophobic membrane of the polymersomes. The absorbance band was attenuated on irradiation with visible light at 530 nm, indicating the reversion of the MC form back to the SP form. However, this transition was much slower than the SP → MC transition due to the increased stability of the MC polymersomes relative to the SP polymersomes. To ensure that the physical integrity of the polymersomes was maintained during the SP ↔ MC switching process, the authors took advantage of cooperative non-covalent interactions such as H-bonding and hydrophobic interactions to stabilise the membranes of the SP polymersomes. In contrast, the emergence of additional zwitterionic and π-π stacking interactions between MC isomers within the bilayer membrane provided extreme stability to the MC polymersomes. These properties were further exploited to investigate the drug release behaviour of the SP and MC polymersomes, using 2′-deoxy-5-fluorouridine (5-dFu) as a model, small, hydrophilic anti-cancer drug. 5-dFu was encapsulated into the hydrophilic cavities of the polymersomes during self-assembly, and the release behaviour of both SP and MC polymersomes was studied. It was found that <10% of 5-dFu was released from the SP polymersomes after 8 h, while ∼90% of the drug was released after 8 h when the polymersomes were first irradiated with UV light for 2 min. The release rate of the drug from the MC polymersomes decreased over time, given the reversion of the MC isomer back to the SP isomer and corresponding reduction of membrane permeability, and the release rate was easily halted by irradiation with visible light. This reversible bilayer permeability was further investigated *in vitro*. The release of the fluorescent DNA intercalating agent 4′,6-diamidino-2-phenylindole (DAPI) from the cavities of the polymersomes in HeLa cells was examined using confocal laser scanning microscopy. As expected, in the absence of UV irradiation, minimal release of the model drug was observed from the SP polymersomes, while on UV irradiation at 405 nm for 2 min, an increase in membrane permeability and efficient release of DAPI was observed. It is clear from these results that the incorporation of SP-based monomers into the bilayers of polymersomes provides a promising route to overcome the issue of poor membrane permeability associated with traditional polymersomes. Their ability to reversibly switch membrane permeability on and off through UV/Vis light triggers also makes them highly attractive for use as controlled and sustained release nanocarriers (Wang et al., 2015).

### Interpenetrating Polymer Networks

Although the self-assembled polymeric systems described above exhibit great promise for site-specific, on-demand drug delivery, particularly with the introduction of the stimuli-responsive SP moieties, there remain several intrinsic limitations that may hinder their successful application *in vivo.* In general, self-assembled nanocarriers, particularly micelles, are unstable and may dissociate in solution, potentially giving rise to unwanted premature release or leaching of the drug from the nanocarrier. The use of biodegradable polymers also means that a burst release is common to these nanocarriers instead of sustained drug release. Additionally, drug release mechanisms typically associated with stimuli-responsive self-assembled polymeric systems, such as stimuli-triggered nanocarrier disassembly, may restrict the ability to halt drug release after the initial application of a release stimulus. Toxic side reactions may also occur due to the release of residual polymeric materials into the cell on nanocarrier disassembly (Soleymani Abyaneh et al., 2015; Venditti, 2019; Yadav et al., 2020).

An alternative to self-assembled polymeric systems are interpenetrating polymer networks (IPNs). IPNs are combinations of two or more polymers in the form of a network, with at least one being synthesised in the presence of another polymer, such that a novel multi-component polymeric system is produced. The properties of the individual polymer network constituents are maintained, allowing for a synergistic and incremental augmentation of the overall properties of the IPN itself. These properties include high stability, biocompatibility and capacity for swelling without loss of structural integrity. Together, they position IPNs as an attractive alternative to self-assembled polymeric nanocarriers (Raina et al., 2019). The beneficial properties of IPNs have been exploited to develop stimuli-responsive DDSs. These DDSs leverage the change in interactions between the polymers, loaded drug molecules and solvent on stimuli application, thus enabling highly controlled drug delivery without disruption to the structure of the IPNs (Ghani et al., 2021a; 2021b).

In a recent paper, Ghani et al. utilised supercritical CO_2_ technology to develop an efficient photo-responsive IPN for reversible and on-demand drug release (Ghani et al., 2021a). The IPN was constructed by impregnating a silicone elastomer with different SP-functionalised guest polymers. A Hansen solubility parameter-based thermodynamic model was used to optimise the polymer composition to enable a high degree of control over drug release. The thermodynamic model worked on the principle that a high work of adhesion between the loaded drug and the guest polymer would reduce premature drug release, as the adsorption of a drug to the guest polymer would be energetically favourable. In contrast, low work of adhesion would result in increased premature release. To test the feasibility of the model, the light-triggered release of a model drug doxycycline from multiple IPNs with different guest polymer compositions was investigated. Firstly, the photochromic properties of the IPNs were examined on exposure to UV and visible radiation, and reversible SP ↔ MC isomerisation was confirmed to be retained in the IPN. Next, the loading of doxycycline into the IPNs was achieved through passive diffusion—the presence of a protic solvent caused the swelling of IPNs by inducing spontaneous and irreversible SP → MC isomerisation, allowing the doxycycline molecules to diffuse into the IPN. Finally, the release properties of several IPNs varying in hydrophilicity were analysed, and the poly (BMA-*co*-HEMA-*co*-SPMA) IPN with 8.5% HEMA was determined to be the optimal IPN for drug delivery. This IPN showed considerable controlled release behaviour, where drug release was increased on irradiation with UV light and decreased or even halted on irradiation with visible light. The hydrophobic BMA and SPMA blocks were found to minimise premature drug release by maintaining a high work of adhesion, as predicted by the model. At the same time, the addition of small amounts of the hydrophilic HEMA allowed for maximal cumulative release in the presence of UV light and inhibition of premature release in its absence. The effect of drug hydrophobicity on the triggered release properties of the IPN was further investigated using five model drugs with increasing hydrophobicity, namely: doxycycline, dopamine, l-dopa, prednisone and curcumin. On irradiation with UV light, 80–90% triggered release was observed for dopamine, l-dopa and prednisone, while triggered releases of ∼60 and 26% were observed for doxycycline and curcumin, respectively. The hydrophilicity of doxycycline accounts for its preference to stay in the loading media rather than adsorb to the IPN. At the same time, the hydrophobicity of curcumin likely caused a reduction in its release from the IPN, in addition to potentially causing its binding to the silicone matrix. Finally, the biocompatibility of the IPN was investigated in human neural stem cells (hNSCs). The IPN was found to have minimal adverse effects on cell viability in the presence or absence of UV light. The IPN was also found to allow successful differentiation of hNSCs into neurons with no morphological damage. Overall, this photo-responsive IPN displayed good light-controlled drug release, with minimal premature release. However, the maximum cumulative release of the model drug doxycycline achievable was still less than those achieved using more hydrophilic IPNs, which were plagued by high premature release (Ghani et al., 2021a).

In an alternative approach, Ghani et al. investigated a SP-photogated IPN-based DDS for on-demand delivery (Ghani et al., 2021b). The IPN was synthesised as follows: firstly, a silicone elastomer prepared using supercritical CO_2_ technology was impregnated with a hydrophilic poly (HEMA-*co*-PEGMEA) hydrogel. Subsequently, carboxyl-containing SP molecules (SPCOOH) were grafted onto the IPN surface. By introducing the photochromic SP onto the IPN surface, rather than as part of the polymer network itself, it was proposed that the SP would instead act as a molecular gate, allowing for high drug loading efficiency while minimising unwanted premature release. In the hydrophobic SP form, the molecular gate would be closed, generating a hydrophobic layer surrounding the IPN and preventing premature drug release. Irradiation with UV light would stimulate the conversion of the SP isomer into the hydrophilic MC isomer, causing the gate to open. This change in hydrophilicity would allow increased diffusion of water molecules into the IPN, causing it to swell and release the loaded drug molecules. Post-synthesis analysis confirmed the successful functionalisation of the hydrogel surface with SPs. The photo-responsive properties of the SP moieties were also successfully retained on surface binding. An investigation of the IPN’s water uptake in the presence and absence of UV light was carried out. It was found that water uptake was higher in the presence of UV light, confirming that when the gate is closed, the hydrophobic SP layer prevents surface wetting. In contrast, on conversion to the hydrophilic MC isomer, water molecules were found to diffuse much more readily into the IPN’s interior. This increase in water uptake in the presence of UV light was, however, dependent on the amount of hydrogel present on the silicone elastomer—the greater the amount of hydrogel present, the lower the increase in water uptake. As with the previous IPN-based DDS (Ghani et al., 2021a), the triggered release of the model drug doxycycline from the SP-photogated IPN was investigated. The IPN was indeed found to effectively inhibit premature doxycycline release in the absence of UV light. This ability to inhibit premature release was, however, found to be dependent on the hydrogel content. IPNs with hydrogel contents of 40 and 50% exhibited very high premature release of doxycycline. In contrast, IPNs with hydrogel contents of 20 and 30% showed much lower premature release. Regardless, all IPNs showed an increase in the cumulative release of doxycycline on UV-irradiation, indicating that the SP molecular gate did indeed work effectively. Although, the cumulative release was significantly less for those IPNs with lower hydrogel contents (20 and 30%) than those with higher hydrogel contents (40 and 50%). These results indicate that the proposed SP-photogated IPN does indeed show promise for on-demand delivery of hydrophilic drugs. However, careful tuning of hydrogel composition is required in order to ensure a compromise is reached between high cumulative drug release and minimal premature release (Ghani et al., 2021b).

### Lipid-Based Nanoparticles

Lipid-based nanocarriers have attracted much attention due to their ability to enhance the solubility and bioavailability of drugs. There are various lipid-based nanocarriers, which are broken down into two categories: vesicular and non-vesicular, such as liposomes and solid lipid nanocarriers, respectively. Lipids enable transport across the mucosal walls of the gastrointestinal tract and protect the encapsulated API from oxidation. The major advantage of incorporating lipids into a DDS is that they are biocompatible and easily broken down by the body (Mihai Grumezescu, 2018). As with other drug carriers, drug release may be unpredictable and systemic rather than local. Thus, there has been an increased interest in developing stimuli-responsive drug release, along with increasing the number of possible doses from a single administration (Timko et al., 2010).

**FIGURE 5:**
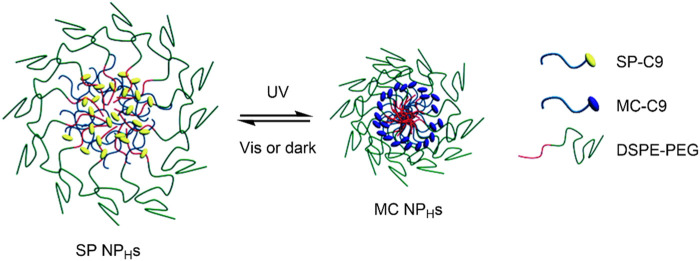
Schematic of the UV-induced shrinkage of the SP NP_H_s system due to SP → MC conversion. Figure adapted with permission from ACS (Tong et al., 2012).

Tong, Kohane et al. described a hybrid SP/lipid-PEG nanoparticle (SP NP_H_s), which was composed of a C9 alkyl chain SP derivative (SP-C9), 1,2-distearoyl-*sn*-glycero-3-phosphoethanolamine-*N*-carboxy (polyethylene glycol)-5,000 (DPSE-PEG), and lecithin (Tong et al., 2012). In solution, the amphiphilic PEGylated lipid underwent self-assembly to form a monodisperse nanoparticle, with the SP-C9 chains composing the hydrophobic core and the PEG chains forming the hydrophilic outer layer. This nanoparticle exhibited light-induced size reduction on irradiation with UV light due to the increase in polarity of the PEGylated lipids on the conversion of the SP NP_H_s to MC NP_H_s (Figure 5). This was reversible in dark conditions or on visible light irradiation. Seven compounds, namely rhodamine B, coumarin 6, cyanine 5, paclitaxel, docetaxel (DTXL), proparacaine, and DOX, were encapsulated in the core of the lipid nanoparticle to show its broad applicability in drug delivery. The toxicity of the unloaded SP NP_H_s was assessed in HeLa, PC-3 and huvec cells, showing minimal cytotoxicity in all cell types at standard concentrations. However, the SP NP_H_s showed photofatigue, with the absorption intensity decreasing after repeated irradiation. This highlighted the potential use of these nanocarriers for delivering multiple dosages from the same administration. The UV-induced shrinkage of the SP NP_H_s, as it was converted into MC NP_H_s, was shown to result in drug release. When the fluorescent molecule calcein was incorporated into the SP NP_H_s system, no fluorescence was observed due to self-quenching as calcein was stored within the nanoparticle. However, upon UV-irradiation (365 nm), strong fluorescence was observed (λ_max_ 510 nm), indicating rapid calcein release from the nanocarrier. This was observed on calcein loaded SP NP_H_s in HeLa cells, strongly suggesting the release of calcein through UV-induced particle size reduction. In the second part of this study, the functionalisation of the SP NP_H_s surface was examined. It was found that when the surface was functionalised with a cell-penetrating protein, the cytotoxicity of DOX loaded SP NP_H_s was significantly increased compared to that of the non-functionalised nanocarrier. This highlights the possibility of using biomolecules to enhance targeted drug delivery in these systems. The group also found that the SP NP_H_s → MC NP_H_s particle size reduction improved drug diffusion through collagen matrices and cornea cadavers, indicating the potential for improved drug penetration and controlled release to tumour cells on target site photo-irradiation (Tong et al., 2012).

In a follow-up paper, the group improved the SP NP_H_s nanocarrier by introducing cholesterol (SP NP_HC_) (Tong et al., 2013). It was shown that the introduction of cholesterol to DTXL loaded SP NP_H_s significantly reduced the release of DTXL in the absence of UV-irradiation. However, drug release remained rapid on irradiation with UV light. The drug release followed the same mechanism described in the previous paper; UV-induced SP NP_H_s → MC NP_HC_ conversion led to particle size reduction and subsequent drug release. It was found that the diffusion of DTXL and its distribution in mice tumour cells was increased upon UV-irradiation of the DTXL loaded SP NP_HC_ nanocarriers. An increased intra-tumoural penetration accompanied this. This is a significant advance, given that the dense matrix present in tumour cells limits the penetration of traditional therapies (Netti et al., 2000). Overall, the nanocarrier showed both spatiotemporal control over drug release and an enhanced tumour penetration on irradiation with UV light, and reduced systemic exposure in the absence of UV-irradiation (Tong et al., 2013).

### Upconversion Nanoparticles

Although light as a stimulus is very convenient, tunable, and offers spatiotemporal control of drug release, the wavelengths required for light-induced drug release from SP-based DDSs limit their applicability. Prolonged exposure to UV light is known to have cytotoxic and mutagenic effects on healthy cells (McMillan et al., 2008). Furthermore, chromophores within bodily tissues, such as haemoglobin and water, have wavelength-dependent extinction coefficients and are strongly absorbing in the UV-Vis region, thus decreasing tissue penetration (Rwei et al., 2015). However, the extinction coefficients of these chromophores are lowest in the NIR region between 650–900 nm; as such, NIR light has a much greater tissue penetrating ability. In addition, NIR light is non-invasive and causes minimal cellular damage (Weissleder, 2001; Ash et al., 2017). Therefore, NIR light is a promising practical alternative to UV-Vis light, having applications in photothermal therapy, stimulus-responsive drug release and *in vivo* deep-tissue imaging (Lim and Park, 2017). However, NIR light does not have enough energy to induce a ring-opening in an SP molecule. One solution to this is two-photon excitation. SP molecules have been shown to isomerise using NIR light due to this effect (Klajn, 2014). However, this may reduce the absorption cross-section for the NIR photons and extend the irradiation time required (Schumers et al., 2010). Alternatively, lanthanide-doped upconversion nanoparticles (UCNPs) have been introduced into DDSs to convert low-energy NIR radiation into higher energy radiation, driving SP ↔ MC isomerisation. The lanthanide group of elements are commonly used in UCNPs as they have partially filled inner 4f orbitals. An electron in inner 4f orbitals may be excited by a low energy photon (NIR) and, given that f-f transitions are formally forbidden, its excited state has a long lifetime; up to several milliseconds (Tu et al., 2015). As a result, a second low-energy photon may be absorbed, and a higher excited state may be reached. Upon relaxation to the ground state, a photon of higher energy than those absorbed is emitted (Bettinelli et al., 2015). This is known as the anti-Stokes (upconversion) process, by which two or more long-wavelength photons are converted into a single photon of a shorter wavelength (Li et al., 2015). Lanthanide dopants commonly used include Yb^3+^, Er^3+^, Tm^3+^, and Ho^3+^ (Lee and Park, 2018). UCNPs are typically hydrophobic, limiting their biomedical application. However, this can be overcome by introducing hydrophilic polymers onto their surface, allowing for a photoresponsive moiety and a drug molecule to be attached to the surface of the UCNP. Alternatively, this can be achieved by forming a nanocomposite, where the UCNPs, drug molecules and SP groups are incorporated into a polymer matrix (Bagheri et al., 2016).

In 2013, Zhou, Qu et al. reported a facile route for the synthesis of hollow NaYF_4_: 18%Yb^3+^/2%Er^3+^ UCNP using DNA without needing a sacrificial template nanoparticle that is commonly required to synthesise UCNPs. It was found that increasing the DNA concentration led to a decrease in particle size, but when less DNA was used, the particles obtained were spherical and hollow, allowing for control of both shape and size (Zhou et al., 2013). To test the loading capacity and photoresponsivity of the UCNPs, MC was immobilised onto their surface (UCNP-MC). The absorption spectra of the UCNP-MC were recorded on irradiation with NIR light at 980 nm, and visible-light-induced MC → SP isomerisation was observed, as facilitated by the upconversion luminescence (λ_em_ 520, 538 and 652 nm) of the UCNPs. Given its charged characteristic, the UCNP-MC form was investigated for both its ability to bind proteins, and its NIR light-triggered release properties, for which β-galactosidase (β-gal) was used as a model enzyme. It was found that the enzyme adsorption to the UCNP-MC surface (UCNP-MC-β-gal) was greatly influenced by the positive charge on the MC groups, with enzyme release only observed as UCNP-MC was converted into UCNP-SP upon NIR irradiation. The system’s *in vitro* behaviour was studied in HeLa cells—the UCNP-MC-β-gal nanocarrier was found to endocytose and accumulate in the cytoplasm. The system showed no cytotoxicity on NIR irradiation, and the NIR-triggered protein release into cells was confirmed. Furthermore, the released enzyme retained its biological activity, thus indicating this nanoparticles’ ability to act as a protein delivery system. This MC-UCNP nanocarrier displayed good spatiotemporal control over protein release, highlighting its potential application beyond protein-delivery for possible light-controlled drug delivery. (Zhou et al., 2013).

In an alternative approach, Chen et al. reported a successful DDS composed of a photo- and pH-responsive SP-functionalised amphiphilic polymer and UCNP based nanocomposite (Chen et al., 2016). In their investigation, a poly (*N*-isopropylacrylamide-*co*-SP methacrylate) amphiphilic block copolymer and a NaYF_4_: 25%Yb^3+^/0.5%Tm^3+^ UCNP were synthesised. In an aqueous solution, the amphiphilic polymers self-assembled into micellar nanoparticles with the *N*-isopropylacrylamide block forming the hydrophilic shell, and the SP-containing methacrylate block forming the hydrophobic core. The hydrophobicity of the micelle core was exploited to encapsulate the UCNPs. The emission spectra of UCNPs on NIR-irradiation (980 nm) were shown to have a λ_max_ at 360 nm, which overlapped with the absorption spectra of the SP groups. Moreover, it was shown that on NIR-irradiation, the absorption band at 525 nm in the nanocomposites’ UV-Vis spectra increased, indicating SP → MC conversion. On irradiation with visible light at 520 nm, the absorption spectra reverted to the original, indicating reversible SP ↔ MC isomerisation in the nanocomposites. Fluorescent coumarin-102 guest molecules were then loaded into the nanocomposites hydrophobic core to investigate their dual photo/pH-controlled encapsulation and release properties. Coumarin-102 release was observed on NIR-irradiation due to the conversion of the hydrophobic SP groups to hydrophilic MC groups, which disrupted the hydrophobic-hydrophilic balance and subsequently caused the micelles to dissociate. Interestingly, low pH conditions led to the swelling of the self-assemblies and subsequent drug release. Reduction in the pH from seven to five and NIR-irradiation led to an increase in coumarin-102 release due to the protonation of the SP to the MC form, indicated by the nanocomposites’ emission spectra. Critically, the nanocomposites were shown to have minimal cytotoxicity *in vitro* on U-87 MG cancer cells, while when loaded with DOX, the nanocomposites caused significant cell death on NIR-irradiation. Overall, these UCNP SP-copolymer nanocomposites show a potential application in anti-cancer treatment—the low-pH tumour environment combined with NIR-irradiation can improve site-specific drug release properties while minimising phototoxic effects (Chen et al., 2016).

### Mesoporous Silica Nanoparticles

Mesoporous silica nanoparticles (MSNs) are solid silica (SiO_2_) frameworks with honeycomb-like porous structures (Pednekar et al., 2017). Mesoporous materials are characterised by pore diameters ranging from 2–50 nm (Rouquerol et al., 1994)—this porous nature of MSNs allows loading and release of therapeutics, with pore size and volume being highly tunable to accommodate a variety of guest molecules. The vast quantity of Si-OH groups present on the MSN surface allows for surface modification with responsive materials, commonly polymers, for controlled-release of therapeutics (Yang et al., 2012; Manzano and Vallet-Regí, 2019). By coating the MSN surface with a hydrophobic polymer, the pores are effectively covered by a hydrophobic layer, thus decreasing the nanoparticle’s wettability and consequently minimising drug release. In contrast, in the presence of a hydrophilic polymer, the surface wettability is increased, which allows for loaded therapeutics to diffuse out of the MSNs (Yang et al., 2012). This process has been exploited by modifying the Si-OH groups with SP-containing polymers. An endo- or exogenous stimuli may induce these to switch from the hydrophobic SP form to the hydrophilic MC form. Thus, allowing for targeted and controlled release of the loaded therapeutic. In addition, MSNs show very good biocompatibility and their size enables efficient cellular uptake by endocytosis, highlighting their potential as nanocarriers for anti-cancer therapeutics (Li et al., 2012; Argyo et al., 2013).

**FIGURE 6:**
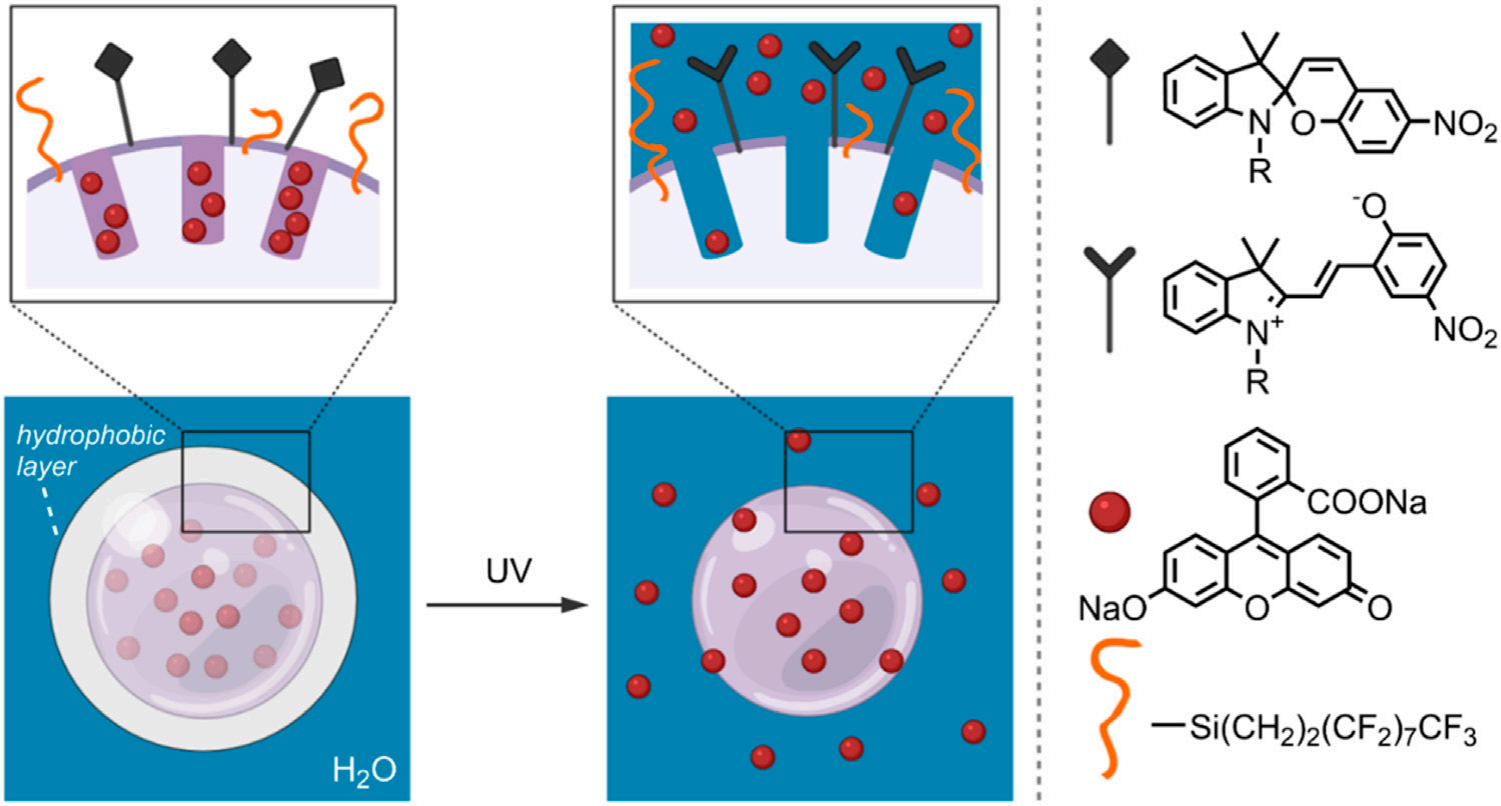
Schematic of an FD-loaded MS-FSP system. On the left, SP groups form a hydrophobic layer, decreasing the wettability of the MSN surface and preventing cargo release. On the right, the hydrophobic SP groups have been converted to the hydrophilic MC form by UV-irradiation. The light-induced increase in surface wettability results in FD release. (Chen et al., 2014).

The ability to control drug release by influencing MSN surface wettability through photo-induced SP isomerisation was investigated by Chen et al., in 2014 (Chen et al., 2014). In their study, 300 nm diameter MSNs were treated with 3-aminopropyltriethoxy-silane (APTES) and perfluorodecyltriethoxysilane (PFDTES). The resulting amine- and fluorinated-silane modified MSNs (MS-FNH_2_) were subsequently functionalised with carboxylic acid-terminated SP, yielding the final hydrophobic DDS (MS-FSP). Using fluorescein disodium (FD) as model cargo, the light-responsive release properties of the MS-FSP were investigated. It was found that rapid release occurred when the surface was functionalised with SP only (MS-SP). However, by increasing the surface hydrophobicity through a high PFDTES: SP ratio, minimal FD release from MS-FSP pores was observed without UV-irradiation—thus, creating a hydrophobic surface layer that effectively prevented undesired FD release. On UV-irradiation, the SP form was converted to the MC form, increasing the MSNs surface hydrophilicity—in this case, FD release was observed (Figure 6). To further investigate the system’s drug-delivery potential, camptothecin (CPT) was loaded into the MS-FSP (MS-FSP-CPT) as a model anticancer drug. The *in vitro* release properties were investigated in EA.hy926 and HeLa cells. After 24 h, minimal cytotoxicity was observed for MS and MS-FSP, with and without a 5 min exposure to UV-irradiation, up to concentrations of 100 μg/ml. Decreased cell viability was observed for both cell types loaded with MS-FSP-CPT, with increased cell death observed when irradiated with UV light, indicating successful light-induced CPT release. Overall, this system has good potential for the controlled release of anti-cancer drugs, having good biocompatibility and photoresponsive characteristics (Chen et al., 2014).

In an alternative approach, Xing et al. examined the drug-delivery feasibility of a hollow MSN (HMSN) coated with an amphiphilic SP-containing copolymer *via* self-assembly (Xing et al., 2014). HMSNs have been increasingly studied for their drug loading ability due to their increased volume for storing therapeutics (Liu et al., 2016). In this investigation, the amphiphilic copolymer was composed of a rhodamine B (2-hydroxyethyl acrylate) ester (RBM) block, a hydrophilic MAPEG block, and a spiropyran methacrylate (SPMA) block. The amphiphilic copolymer, labelled PRMS, was then conjugated with folic acid (FA), which is known to target folate receptors (FR) in tumour cells (Fernández et al., 2018). The absorption bands of the SPMA at 365 nm were found to overlap well with the emission bands of the RBM. Thus, a FRET process could be utilised, with RBM acting as the donor and SPMA acting as the acceptor. This was indeed observed as the fluorescent intensity of the rhodamine B moiety, excited at 510 nm, significantly decreased on irradiation with UV light at 365 nm. This was a result of the conversion of the closed SP form to the open, visible light absorbing MC form, leading to the quenching of the rhodamine B fluorescence. The polymer was then coated onto the surface of an HMSN modified with C-18 alkyl chains by self-assembly through hydrophobic interactions between the C-18 chains and the polymer’s hydrophobic blocks. The coating process was shown to block the pores of the HMSN by forming a hydrophobic layer at the HMSN surface. The HMSN/C-18/PRMS-FA nanocarrier’s controlled-release properties were investigated, using DOX as a model therapeutic. The amphiphilic polymer on the HMSN surface effectively blocked the pores, preventing the release of DOX in the absence of UV and visible light, and minimising the quantity released after 8 h. Rapid release was observed with UV-irradiation, with 70 wt% of DOX released in 100 h, and the initial rapid release was found to halt immediately upon switching to a visible irradiation source. Moreover, the drug release was found to co-occur with the FRET process, allowing for real-time monitoring of the DOX levels in the HMSNs. The HMSN/C-18/PRMS-FA nanocarriers showed good biocompatibility and good targeting in FR containing tumour cells compared to non-FR containing tumour cells due to the FA moiety in the polymer. Based on these results, this system shows promise for its biomedical applications; particularly, the targeted and controlled release of anti-cancer drugs to FR containing tumour cells (Xing et al., 2014).

Although MSNs have many benefits, they have been shown to have low renal clearance and poor biodegradability (Croissant et al., 2017). In addition, the limitations associated with using UV light, as previously discussed (*Polymersomes* Section), are pertinent. In light of this, He et al. recently proposed a system that addresses both of these issues (He et al., 2020). The system was composed of an SP and fluorinated silane (FS) modified ultrasmall mesoporous silica nanoparticle (SP-FS-USMSN; approx. 12 nm diameter)—the FS was used to increase surface hydrophobicity. In a pH 7.4 PBS solution, the SP-FS-USMSN self-assembled through hydrophobic interactions into nanoclusters (approx. 110 nm diameter). The clusters were shown to dissociate at pH 4.5 and 5.5—the low pH caused the hydrophobic SP form to convert to the hydrophilic MC form, thus resulting in cluster dissociation. These nanoclusters were used for co-delivery of DOX and curcumin (CUR). DOX was loaded into the pores of SP-FS-USMSNs by electrostatic interactions with the surface silanol groups. The DOX-loaded SP-FS-USMSN were then allowed to assemble *via* hydrophobic interactions with CUR, to form DOX and CUR-loaded SP-FS-USMSN nanoclusters. The systems release properties were then studied, and a significant increase in drug release was observed at pH 5.5 and 4.5 for both DOX and CUR. This low-pH-induced release is a contrast to the decreased release observed at pH 7.4 and 6.5. Through *in vitro* studies, the nanoclusters were shown to exhibit significant cellular uptake in HepG2 cells due to the EPR effect. Through DOX and CUR fluorescence monitoring, the drugs were shown to be widely distributed in HepG2 cells after 8 h. This desirable biodistribution is believed to result from the low-pH environment of endosomes, causing the SP → MC conversion-induced disassociation of the nanoclusters and subsequent release of the loaded therapeutics. The *in vitro* cytotoxicity of DOX- and CUR and DOX-loaded SP-FS-USMSN nanoclusters was also investigated. The dual loaded CUR-DOX-SP-FS-USMSN displayed a higher cytotoxicity than that of the DOX-loaded SP-FS-USMSN, indicating the combinatorial anti-tumour effects of CUR and DOX. The SP-FS-USMSN cluster, on the other hand, showed no inhibitory effect on cell viability, indicating its biocompatibility. HepG2-xenografted nude mice were used to determine the *in vivo* properties of the system. DOX and CUR loaded SP-FS-USMSN showed a 74% tumour growth inhibition rate and were found to cause dramatic cell shrinkage and necrosis. Further *in vivo* studies in mice showed large amounts of silica present in both urine and faeces, indicating that the USMSN could be rapidly cleared from the body upon disassociation of the clusters at low pH. This DDS shows promise for their use in anti-cancer therapy for its considerable accumulation in tumour cells, the potential for effective co-delivery of complementary therapeutics, its rapid clearance from the body, and the fact that harmful UV-irradiation is not required to induce drug-release in this system (He et al., 2020).

### SP-Metal Ion Complexes and Carbon Nanomaterials

**FIGURE 7:**
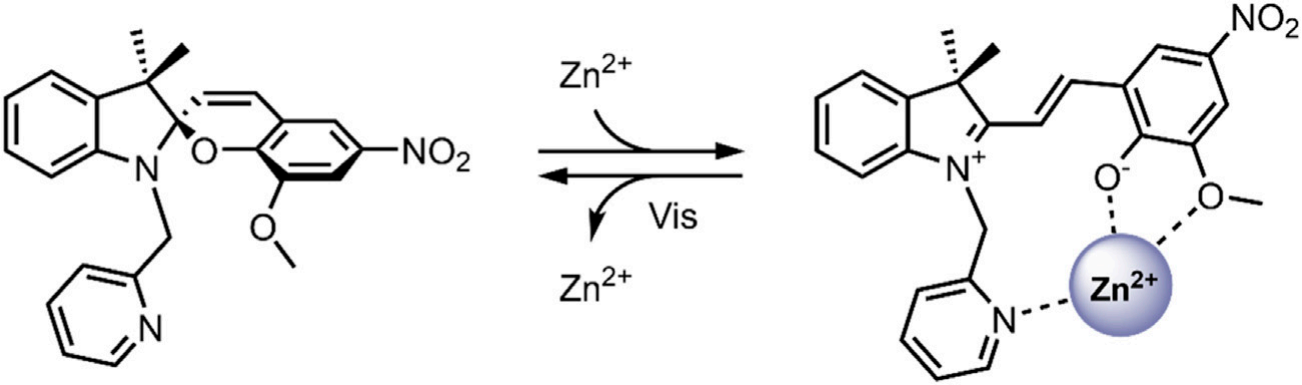
Schematic of the photo-reversible SP → MC-Zn^2+^ complex formation (Natali et al., 2010b).

The attention given to the complexation of SPs with metal cations has been increasing in the scientific community over recent years (Natali et al., 2010a; Del Canto et al., 2012; Heng et al., 2018; Cardano et al., 2019). The planar, open MC form contains an anionic phenolate shown to reversibly chelate to electron-poor metal ions differing in softness/hardness, such as Zn^2+^ and Cu^2+^, with optical control (Figure 7) (Phillips et al., 1965; Natali and Giordani, 2012a). Also, functionalisation of the benzopyran and indoline moieties with additional chelating groups can influence the metal ion-spiropyran complex stoichiometry and geometry without significantly affecting the SP ↔ MC isomerisation (Görner and Chibisov, 1998; Natali and Giordani, 2012b; Klajn, 2014; Baldrighi et al., 2016). The on/off switching ability of SP-metal ion complexation has found many biomedical applications, particularly in biosensors (Radu et al., 2009; Ali et al., 2020). More recently, the potential of SPs has been exploited for the stimuli-controlled ion and drug release from nanocarriers (Del Canto et al., 2012; Heng et al., 2018; Cardano et al., 2019).

**FIGURE 8:**
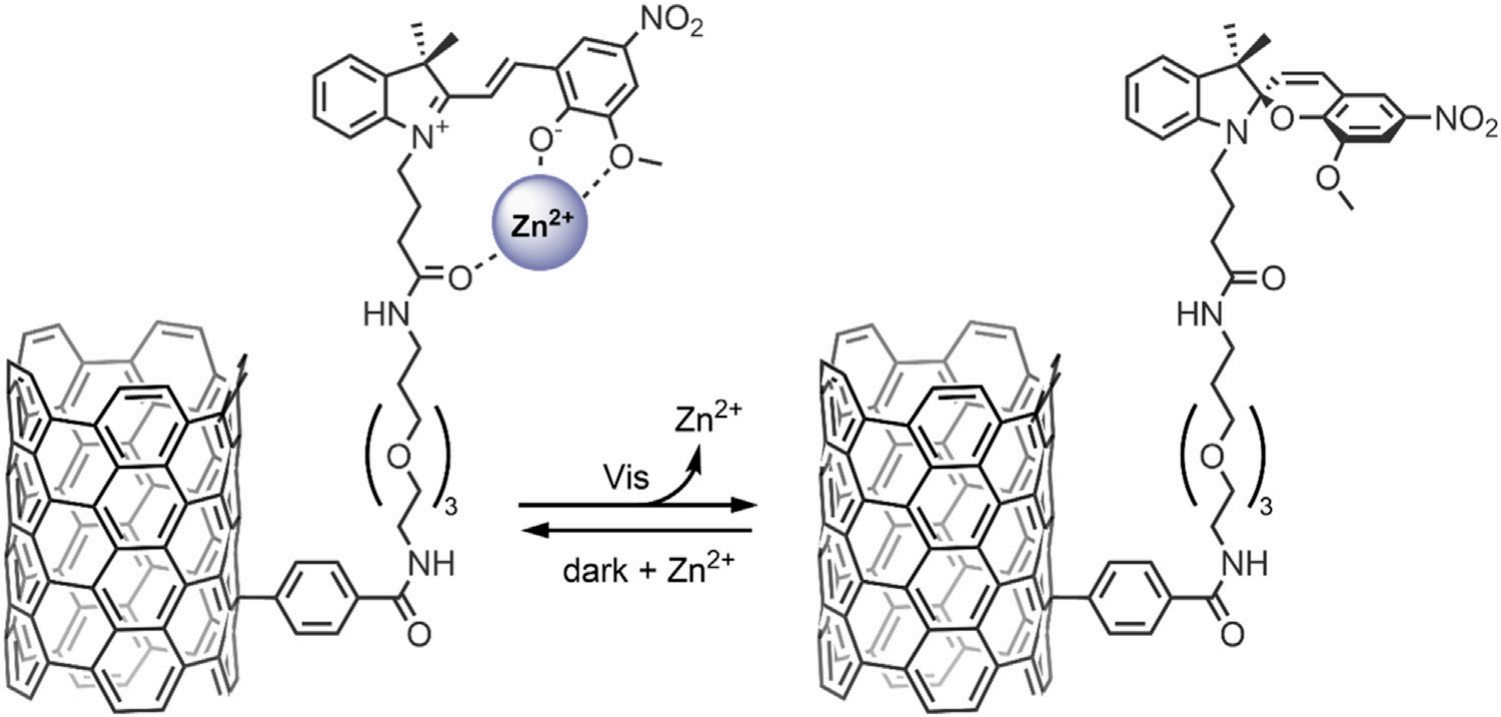
Light-induced MC → SP conversion in the functionalised SWNT nanocarrier, resulting in the release of Zn^2+^ ions from the MC molecules anchored to the SWNT surface (Del Canto et al., 2012).

Carbon nanomaterials (CNMs) are chemically stable, and their surfaces can be easily modified, allowing for robust control of their physicochemical properties and functions within the body—as such, they lend to many biological applications, especially in the case of photoresponsive CNMs (Cardano et al., 2018). In particular, single-walled carbon nanotubes (SWNTs) and graphene show potential for their use in light-controlled DDSs, when modifying their surface with photochromic SP molecules (Del Canto et al., 2012; Nahain et al., 2013). It has been shown that when bound to the surface of these nanomaterials, SPs retain their ability to bind to metal ions and have shown SP → MC isomerisation when exposed to Zn^2+^ (Del Canto et al., 2010; Perry et al., 2015). These findings have been exploited to design smart SP-functionalised SWNTs for the light-controlled release of Zn^2+^ (Del Canto et al., 2012). In our 2012 study, we investigated the ability of an SP bound to the surface of a SWNT as a receptor to reversibly uptake Zn^2+^ in response to light (Figure 8) (Del Canto et al., 2012). The SP molecules were bound to the SWNT surface *via* a PEG linker, which was used to enhance biocompatibility and promote the nanocarrier’s renal clearance. The SWNTs were first purified to remove the toxic metal catalysts and then covalently modified using a Tour reaction to introduce benzoic acid moieties, which further improved the nanocarrier’s dispersibility. The benzoic acid moieties were then coupled with a PEG linker and the SP derivative *via* an amidation reaction. The absorbance maximum of SP-SWNT (λ_max_ 416 nm) was redshifted relative to that of free SP in solution, confirming the successful functionalization of the surface of the SWNT. An absorption maximum at 585 nm was obtained after UV-irradiation (365 nm for 2 min), which disappeared after 3 min in the dark, indicating reversible SP ↔ MC isomerisation. Emission spectroscopy was used to confirm the formation of the MC-Zn^2+^ complex, and light/darkness cycles showed the sequential uptake and release of Zn^2+^ by the SWNT-anchored SP molecules. These findings highlight the potential of SP/SWNT-based systems to be used for drug delivery, where the photo-controlled switching of SWNT-anchored SP molecules may modulate the release of bound therapeutic agents at target locations (Del Canto et al., 2012).

**FIGURE 9:**
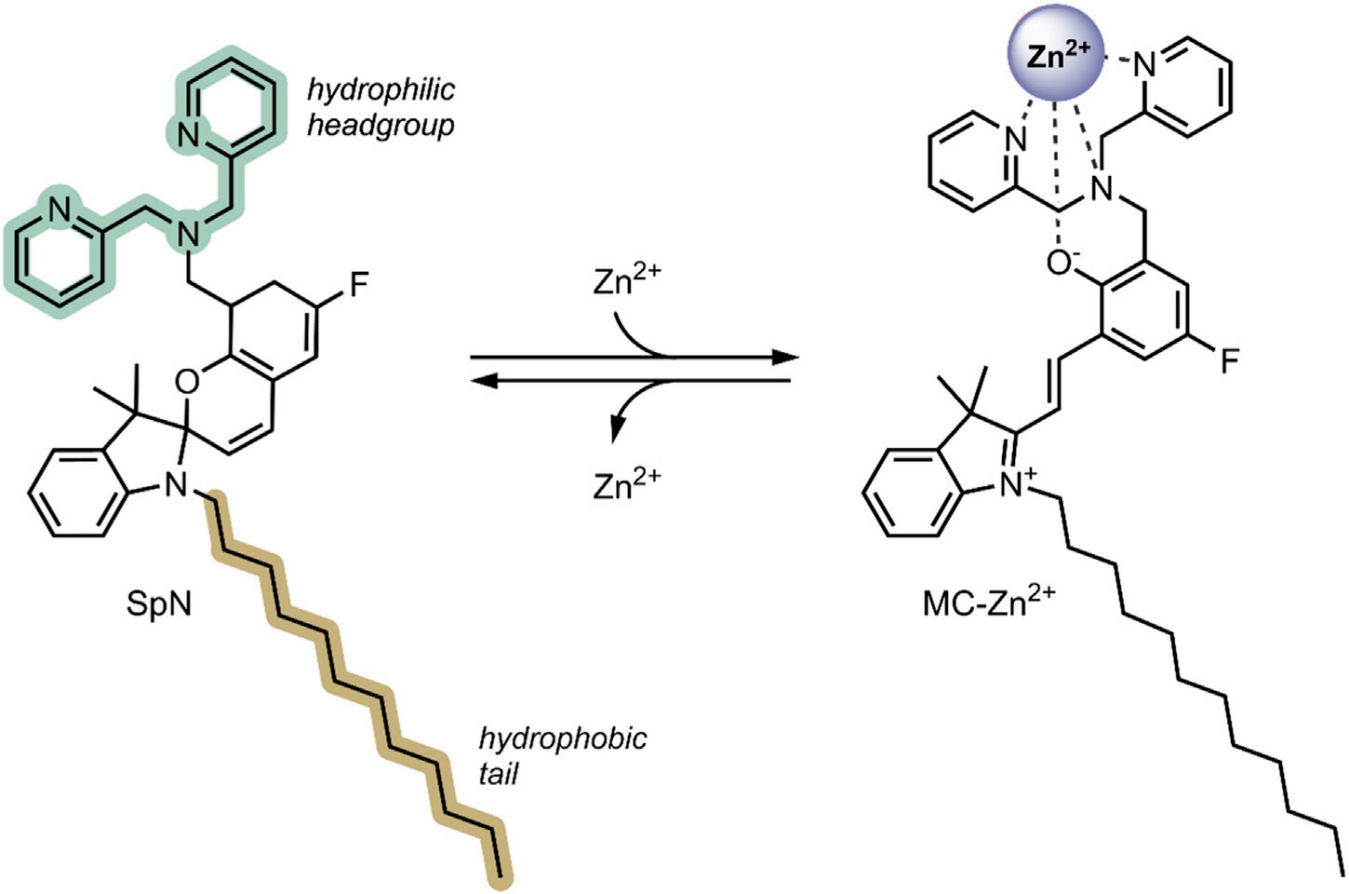
The structure of the SpN is shown on the left. The presence of high levels of Zn^2+^ in tumour cells induces a ring-opening and formation of a fluorescent MC-Zn^2+^ complex, shown on the right. The three nitrogen atoms in the bis(2-pyridylmethyl)amine group and the phenolate oxygen were involved in chelating to the Zn^2+^ ion (Heng et al., 2018).

In 2018, Heng et al. reported a Zn^2+^ responsive SP-based nanocarrier (Heng et al., 2018). Some tumour cells have elevated Zn^2+^ levels compared to healthy cells—these Zn^2+^ levels tightly regulate the activity of specific caspases, which are known to induce apoptosis (Brown and Attardi, 2005). Thus, the reported system took advantage of the favourable conversion of the SP form to the MC form in the presence of high levels of Zn^2+^, resulting in the swelling of the nanocarriers and the release of encapsulated therapeutic. The system (SpN) was composed of an SP molecule containing a hydrophobic C-12 substituent on the nitrogen of the indolenine moiety and a hydrophilic bis(2-pyridylmethyl)amine group on the benzopyran moiety (Figure 9). The SpN self-assembled in an aqueous solution to form a micelle-like nanocarrier of an average diameter of 575 nm. The particle diameter was found to increase linearly with increasing Zn^2+^ concentration, owing to the SP form switching to the fluorescent MC-Zn^2+^ complex. Using the fluorophores 7-hydroxycoumarin and 1-hydroxypyrene, the nanocarriers’ encapsulation and release properties were investigated in apoptotic and healthy HEK293 cells. In apoptotic HEK293 cells, elevated levels of Zn^2+^, intense fluorescence, and distribution in the cells were observed for both fluorophores. This was not the case in healthy cells. This indicated that in the absence of Zn^2+^, the molecules remained trapped in the nanocarriers, but are released in the presence of Zn^2+^ due to complex formation and swelling of the nanocarrier. The fluorescent intensity increased linearly with increasing Zn^2+^ concentration, highlighting potential use in real-time Zn^2+^ sensing. The nanocarriers were further used to encapsulate and deliver Azure B, a caspase inhibitor, into apoptotic HEK293 cells and a potent proteasome inhibitor to breast cancer cell lines, T46D and MDA-MB-468. Caspase inhibition by Azure B released from the SpN was observed, validated by time-lapsed cell microscopy. Significant cytotoxicity was observed in the breast cancer cell lines due to the release of the proteasome inhibitor in the presence of Zn^2+^. No cytotoxicity was observed in the absence of Zn^2+^. Both the Azure B and proteasome inhibitor’s therapeutic activity depended on Zn^2+^ concentration, where increased inhibition was observed with increasing Zn^2+^ concentration. The Zn^2+^ responsive nature of this system enabled targeted release of anti-cancer therapeutics in tumour cells, limiting systemic exposure, and the fluorescent nature of the complex formed enabled real-time analysis of the Zn^2+^ concentration, indicating early or late-stage apoptosis (Heng et al., 2018).

**FIGURE 10:**
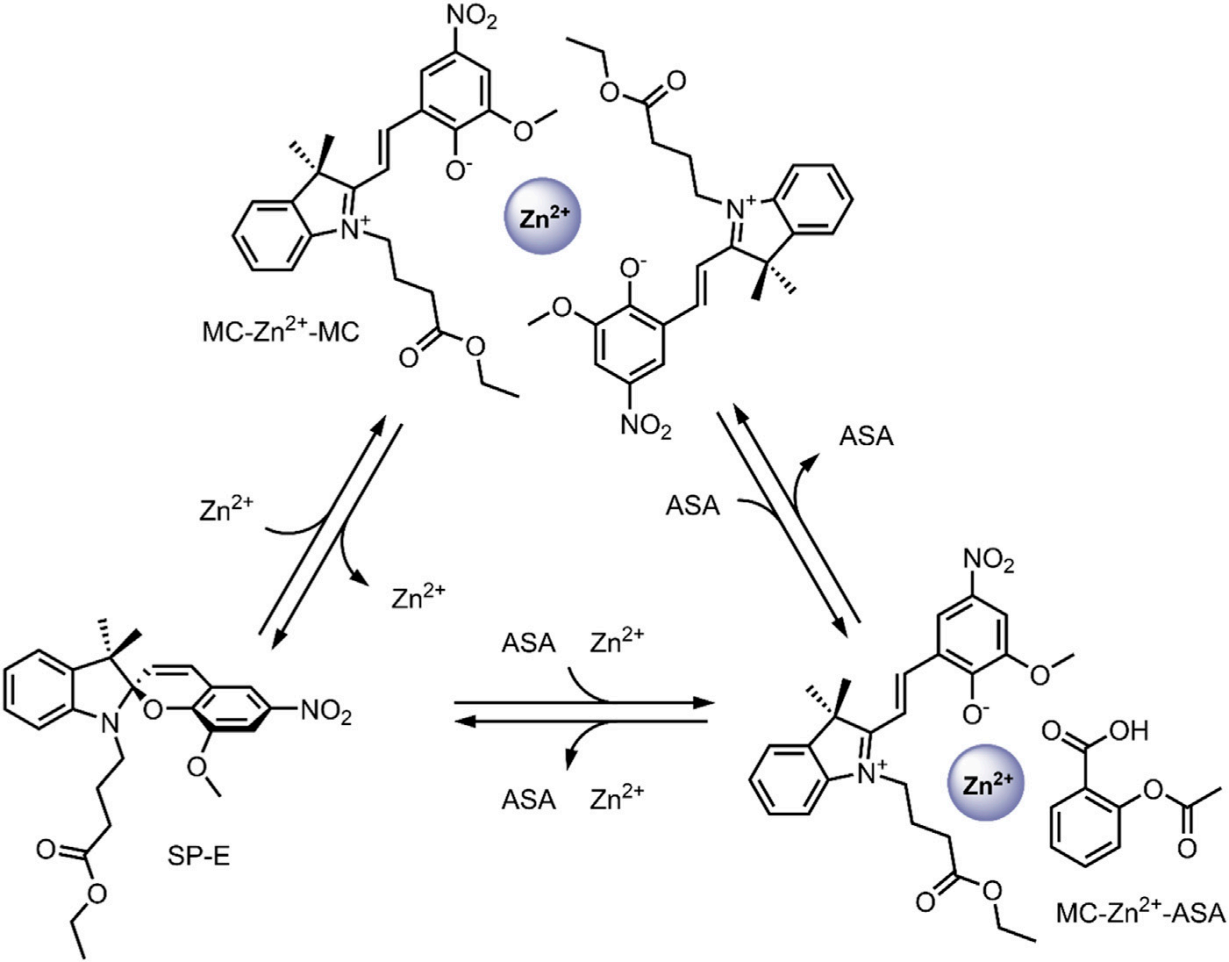
Schematic representation of the formation and dissociation of the MC-Zn^2+^-ASA and MC-Zn^2+^-MC ternary complexes (Cardano et al., 2019).

In 2019, our group probed the use of modified SPs for dual co-delivery applications (Cardano et al., 2019). For this study, a Zn^2+^ and acetylsalicylic acid (ASA) pair was chosen as model cargo, given their excellent anti-inflammatory activity (Prasad, 2014; Jarosz et al., 2017). A spiropyran ester (SP-E) derivative was synthesised in our previous work, and its interactions with divalent metal cations (Zn^2+^, Cu^2+^, and Mg^2+^) were extensively studied—XRD analysis indicated that the metal cations chelate the SP-E molecule’s through their phenolate and methoxy moieties (Baldrighi et al., 2016). Our study found that the SP-E forms a stable ternary complex in the MC form with Zn^2+^ and ASA (Figure 10). The ternary complex formation was found to occur only when the components were added without any pre-complexation, and the ternary complex was stable for 1 month. The system’s photo-responsiveness was then investigated—the conversion of the SP-E moiety from ring-opened MC form to the ring closed SP form was observed on visible light irradiation, leading to the release of Zn^2+^ and ASA. The ternary complex was found to reform within 2 h in the dark. The ability to switch the SP-E moiety between open/closed forms on Vis-irradiation highlights its potential as a smart DDS for light-controlled co-delivery of anti-inflammatory agents to areas of the body with direct exposure to visible light; such as the skin (Cardano et al., 2019). Prospectively, the development of optical fibres may allow for this ternary DDS to co-deliver therapeutics to targets within the body which are not penetrable by visible light (Keiser, 2019).

## Conclusion

The photochromic behaviour of spiropyrans (SP) has been extensively studied since it was first observed in the 1950s by Fischer and Hirshberg. On the application of a stimulus, most commonly light, the SP form undergoes a ring-opening to a physically and chemically distinct merocyanine (MC) form, with a corresponding redshift in the UV-Vis spectrum. With the extraordinary advances in pharmaceutical and materials science, there has been a resurgent interest in the ability of SPs to switch between two distinct, stable isomers, lending to their biomedical applications, particularly in biosensing and nano-based drug delivery. The present review highlights several promising SP-based nanocarriers that leverage stimulus-induced SP → MC conversion for site-specific and dose-controlled release of drugs. In particular, SPs have found successful incorporation into polymeric micelles, lipid nanoparticles, upconversion nanoparticles (UCNPs), and mesoporous silica nanoparticles (MSNs), as a result of their broad-range compatibility with nanomaterials. These systems show good biocompatibility, bioavailability, and the versatility of the nanomaterials used allows for their release properties to be tuned such that minimal drug release is observed in the absence of a stimulus, but is rapid on stimulus application. Also, by incorporating a second chromophore into the systems, the Förster Resonance Energy Transfer (FRET) mechanism may be exploited for real-time quantification of drug release. SP-based nanocarriers also show responsivity to a variety of endogenous and exogenous stimuli, such as pH, heat and light. Due to its spatiotemporal control over drug release, light is the most commonly used stimulus in SP-based drug-delivery systems (DDSs). However, due to the well-known mutagenic effects of UV light and its insufficient penetration power through bodily tissues, its biomedical applications are limited. This is overcome by using longer wavelength NIR light, which may be ‘upconverted’ into higher-energy light capable of driving the SP → MC conversion, thus releasing the loaded therapeutic. This process can be achieved through the use of lanthanide-doped UCNPs. In an alternative approach, divalent metal ions, particularly Zn^2+^, can be used to induce SP ↔ MC isomerisation, with the MC form favourably forming MC-Zn^2+^ complexes. Overall, spiropyrans prove to be a versatile material that shows promise in light-controlled drug delivery.

The need to move beyond traditional therapies to achieve greater control over drug delivery drives the continued development of responsive materials and smart DDSs. A large focus of SP-based DDS research has been placed on optimising the fabrication of nanocarriers to ensure biocompatibility, high drug loading efficiency, and effective release in the presence of a stimulus, while minimising premature release. However, very little *in vivo* preclinical data has been reported thus far. While biocompatibility will remain of utmost importance, several challenges must be overcome to advance the field toward successful clinical outcomes. Firstly, many of the DDSs developed, particularly self-assembled systems, typically undergo irreversible disassembly/disintegration on the application of a release stimulus. This limits the number of doses that can be delivered with a single administration to one and prevents the halting of drug release after the initial stimulus. Drug delivery vectors such as IPNs and MSNs do not suffer from this issue and may offer a promising alternative to self-assembled systems. Additionally, the excellent physicochemical properties of CNMs have led to their potential use as efficient DDSs, as successfully explored in recent years (Pattnaik et al., 2020). Nevertheless, the research on the introduction of SPs into these IPN, MSN and CNM-based DDSs for triggered, on-demand drug delivery is still in its infancy, with further work required to determine their clinical viability. A second factor to consider is the choice of drug-release stimuli. Although UV-light is the most effective stimulus, the drawbacks in a clinical setting are well-known. The use of NIR light instead offers the obvious benefits of enhanced penetration and reduced cellular damage. However, to achieve the desired effect (SP ↔ MC isomerisation) through two photon NIR excitation, much longer irradiation times are required. For this reason, UCNPs are commonly utilised for NIR to UV light upconversion. However, the health effects of UCNPs are still not well understood (Gnach et al., 2015). The use of different endogenous stimuli, such as temperature, pH and metal ions, has also been investigated to some success. However, their inherent variability *in vivo* makes it difficult to achieve precise control over drug delivery.
